# Diagnostic Terminology and Clinical Practice in Interstitial Ectopic Pregnancy: A Five-Year Clinical Audit

**DOI:** 10.7759/cureus.117724

**Published:** 2026-10-10

**Authors:** Sumi M Surendran, Irina Ramzan, Segun Oniyide, Supreet Mahurkar

**Affiliations:** 1 Obstetrics and Gynaecology, Aneurin Bevan University Health Board, Newport, GBR

**Keywords:** clinical audit, cornual pregnancy, diagnostic terminology, early pregnancy assessment unit, interstitial ectopic pregnancy, transvaginal ultrasonography

## Abstract

Background

Interstitial ectopic pregnancy is rare and carries risks of rupture and severe haemorrhage. Accurate terminology is important because *interstitial* and *cornual* pregnancy are often used interchangeably despite representing distinct implantation sites. The primary objective of this five-year audit was to assess compliance with anatomically precise diagnostic terminology. Secondary objectives evaluated ultrasound assessment, escalation of diagnostically uncertain findings, and documented individualisation of management.

Methods

We retrospectively audited confirmed cases diagnosed between October 2020 and December 2025. Cases identified from Early Pregnancy Assessment Unit clinical and ultrasound records were verified against recognised diagnostic criteria. Five predefined criteria based on the National Institute for Health and Care Excellence (NICE), Royal College of Obstetricians and Gynaecologists (RCOG), and European Society of Human Reproduction and Embryology (ESHRE) guidance assessed transvaginal ultrasonography, compatible anatomical features, escalation of uncertainty, precise terminology in the final ultrasound conclusion, and individualised management. The target was 100% for each applicable criterion. Cases were classified as compliant, non-compliant, or not assessable and analysed descriptively.

Results

Five cases were identified. All patients underwent transvaginal ultrasonography, with reports documenting anatomical findings compatible with interstitial ectopic pregnancy. Across the five reports, these included an empty uterine cavity, a laterally located gestational sac, a thin surrounding myometrial mantle, and an interstitial line sign. Diagnosis was established on the first scan in three cases, while two diagnostically uncertain cases were confirmed following repeat assessment and lead-sonographer review. All pregnancies were diagnosed before rupture and before eight weeks’ gestation. However, all final ultrasound conclusions used the term *confirmed cornual ectopic pregnancy*, resulting in 0% compliance with anatomically precise terminology. Management was individualised and included methotrexate, hysteroscopic resection, and surgery initiated laparoscopically. No major complications were recorded, and all hospital stays were two days or fewer.

Conclusions

The principal finding of this five-case retrospective audit was 0% compliance with anatomically precise terminology: all final ultrasound conclusions used the term *cornual ectopic pregnancy* despite imaging findings compatible with interstitial ectopic pregnancy. This represents a terminology and documentation gap rather than evidence of deficient diagnostic recognition or clinical management. Conversely, compliance was 100% for transvaginal ultrasound assessment, documentation of compatible anatomical features, escalation of diagnostically uncertain findings, and documented individualisation of management. Favourable short-term outcomes were descriptive observations and do not demonstrate the effectiveness or superiority of the diagnostic pathway or any management strategy. Standardised anatomical nomenclature may improve clinical communication, documentation quality, coding accuracy, audit reliability, and future research reporting. Educational initiatives and standardised reporting templates should reinforce the distinction between interstitial, cornual, and angular pregnancies, with re-audit recommended following improvement measures are implemented.

## Introduction

Interstitial ectopic pregnancy is a rare form of ectopic pregnancy occurring when implantation takes place within the interstitial portion of the fallopian tube, the proximal segment traversing the uterine myometrium. Accurate classification of pregnancy location is important because different types of ectopic pregnancy are associated with distinct anatomical characteristics, management considerations, and clinical outcomes [1].

Historically, the terms "interstitial pregnancy" and "cornual pregnancy" have frequently been used interchangeably despite describing different anatomical entities. According to current European Society of Human Reproduction and Embryology (ESHRE) recommendations, interstitial pregnancy refers to implantation within the intramural portion of the fallopian tube, whereas cornual pregnancy should be reserved for pregnancies developing within a rudimentary uterine horn or within the horn of a congenital uterine anomaly such as a bicornuate uterus [1].

Interstitial ectopic pregnancy accounts for approximately 2-6.8% of all ectopic pregnancies and is associated with substantial maternal morbidity because the surrounding myometrium permits progressive enlargement before rupture occurs. Delayed diagnosis may therefore result in significant haemorrhage and increased risk of adverse outcomes [2,3].

Angular pregnancy represents a separate entity that may create additional diagnostic uncertainty. Unlike interstitial pregnancy, angular pregnancy occurs within the endometrial cavity medial to the uterotubal junction, whereas interstitial pregnancy occurs within the myometrial portion of the fallopian tube. Cornual pregnancy, by contrast, occurs within a malformed uterine horn. These conditions differ significantly in anatomical location, prognosis, and management strategy, highlighting the importance of precise terminology and accurate ultrasound diagnosis [1,4-6].

Transvaginal ultrasonography remains the principal diagnostic modality for the localisation of ectopic pregnancy. Recognised ultrasound findings suggestive of interstitial pregnancy include an empty uterine cavity, a gestational sac located laterally within the interstitial portion of the tube, a thin surrounding myometrial mantle, and the presence of an interstitial line sign [2,3,7]. Contemporary guidance from the Royal College of Obstetricians and Gynaecologists (RCOG) and the National Institute for Health and Care Excellence (NICE) recommends prompt diagnosis, escalation of diagnostically uncertain cases, and individualised management based on clinical findings, serum β-hCG concentrations, and patient-specific factors [3,6].

Despite published recommendations supporting standardised terminology, confusion between interstitial and cornual pregnancy continues to be reported in clinical practice and within the literature [1,4,5]. Inaccurate terminology may contribute to inconsistency in clinical documentation, audit activity, coding, and research reporting.

The primary objective of this clinical audit was to assess compliance with anatomically precise diagnostic terminology in confirmed interstitial ectopic pregnancy. Secondary objectives were to evaluate compliance with the recommended ultrasound assessment, escalation of uncertain findings, and documented individualisation of management. By comparing local practice against predefined standards derived from ESHRE, RCOG, and NICE guidance, this audit aimed to identify opportunities for quality improvement in the diagnosis, reporting, and management of this rare but potentially life-threatening condition [1,3,6].

## Materials and methods

Study design and case identification

A retrospective clinical audit was conducted within the Early Pregnancy Assessment Unit between October 2020 and December 2025. Cases were identified through review of Early Pregnancy Assessment Unit clinical records, ultrasound databases, and ectopic pregnancy documentation. To minimise the risk of case omission due to inconsistent nomenclature, all cases documented as either interstitial ectopic pregnancy or cornual ectopic pregnancy were reviewed individually. Five cases met the inclusion criterion of documented ultrasound findings consistent with recognised diagnostic features of interstitial ectopic pregnancy, as described by ESHRE recommendations and RCOG guidance [1,3]. Cases without sufficient documented imaging findings to verify the pregnancy location were excluded from the audit cohort.

Data collection

Data were extracted retrospectively from electronic clinical records, ultrasound reports, laboratory records, and operative documentation. Variables collected included maternal age, presenting symptoms, gestational age at diagnosis, serum β-hCG concentration, ultrasound findings, management approach, operative details where applicable, complications, length of hospital stay, and clinical outcomes.

Extracted data were reviewed against source documentation to verify consistency and completeness. Each audit criterion was classified as compliant, non-compliant, or not assessable for each case. A criterion was recorded as not assessable when the information required to determine compliance could not be identified in the available records; these cases were excluded only from the denominator for that criterion.

Audit criteria and target standards

Five audit criteria were established prior to data collection using recommendations from ESHRE guidance, RCOG Green-top Guideline No. 21, and NICE Guideline NG126 [1,3,6].

Criterion 1

The use of transvaginal ultrasonography as the primary diagnostic imaging modality.

Criterion 2

The documentation of anatomical ultrasound findings compatible with interstitial ectopic pregnancy. Recognised ultrasound features included an empty uterine cavity, a gestational sac located laterally within the interstitial portion of the fallopian tube, a thin surrounding myometrial mantle (<5 mm), and the presence of an interstitial line sign. These features are recognised diagnostic indicators of interstitial pregnancy within contemporary ultrasound literature and national guidance [2,3,7].

Criterion 3

Appropriate escalation of diagnostically uncertain findings. Cases were considered uncertain when the initial ultrasound findings were insufficient to confidently establish pregnancy location and therefore required repeat ultrasound assessment, senior sonographer review, or further specialist evaluation before a final diagnosis could be confirmed [1,3].

Criterion 4

The use of anatomically precise terminology in the final ultrasound conclusion. Terminology was assessed against contemporary ESHRE definitions distinguishing interstitial, cornual, and angular pregnancies [1].

Criterion 5

Documented individualisation of management. Management was considered individualised when documentation demonstrated consideration of patient symptoms, haemodynamic status, ultrasound findings, serum β-hCG concentration and trends, fertility considerations, haemorrhage risk, and available local expertise in determining the most appropriate management strategy [3,6,8-10].

A target compliance rate of 100% was selected for all audit criteria because the audit focused on standards considered fundamental to accurate diagnosis, safe clinical decision-making, clear communication, and high-quality documentation in a potentially life-threatening condition [3,6].

Data analysis

Compliance was calculated by dividing the number of compliant cases by the number of assessable cases and multiplying by 100.

$\text{Compliance (\%)} = \left( \frac{\text{Number of compliant cases}} {\text{Number of assessable cases}} \right) \times 100$

Data were summarised descriptively using counts, percentages, medians, and ranges in Microsoft Excel for Microsoft 365 MSO (Version 2608, Build 16.0.20326.20072; 64-bit; Microsoft Corporation, Redmond, Washington). No inferential statistical analyses were performed because only five cases were included.

Governance and ethics

The project was registered with the Obstetrics and Gynaecology Audit Department before data analysis. As this was a retrospective clinical audit of routinely collected clinical data, formal research ethics approval was not required under local governance arrangements. All patient data were de-identified before analysis, and no patient-identifiable information was included in the manuscript.

## Results

Patient characteristics and presentation

Five women with interstitial ectopic pregnancy were identified. The median age was 32 years (range 27-35), and 5 (100%) patients were diagnosed before eight weeks’ gestation. Abdominal pain was reported in 3 (60%) patients, including shoulder-tip pain in 1 (20%); vaginal bleeding was reported in 1 (20%), and 1 (20%) patient was asymptomatic. Serum β-hCG concentrations ranged from 2,716 to 81,078 IU/L; all 5 (100%) values were above the unit’s transvaginal-ultrasonography discriminatory level of 1,500 IU/L. Patient demographics and presenting features are summarised in Table 1.

**Table 1:** Patient Demographics and Presentation

Patient	Age	Parity	Symptoms	Gestational Age (Weeks)	β-hCG (IU/L)	Past Surgical History
A	27	2	PV bleeding	5.4	2,716	Previous caesarean section
B	34	1	Abdominal pain	5.8	81,078	None
C	31	0	Abdominal pain	6.0	27,000	Laparoscopic appendicectomy; laparoscopy for endometriosis
D	35	2	Abdominal pain and shoulder-tip pain	6.6	40,000	Caesarean section and ovarian cystectomy
E	32	1	Asymptomatic	7.0	10,000	Intrauterine system in situ

Ultrasound findings

Transvaginal ultrasonography was performed in all five patients (100%) by experienced sonographers. All ultrasound reports documented anatomical findings compatible with interstitial ectopic pregnancy. Across the five reports, these findings included an empty uterine cavity, a laterally located gestational sac, a thin surrounding myometrial mantle and an interstitial line sign.

Diagnosis was established at the initial ultrasound assessment in three cases (60%; Patients A, C, and E). In the remaining two cases (40%; Patients B and D), the initial findings were considered diagnostically uncertain, prompting repeat assessment and review by the lead sonographer before a definitive diagnosis was confirmed. All five pregnancies (100%) were diagnosed before rupture, and all patients remained clinically stable at the time of diagnosis.

Although the imaging findings were compatible with interstitial ectopic pregnancy in every case, the final ultrasound conclusion recorded “confirmed cornual ectopic pregnancy” in all five cases (100%). Consequently, compliance with imaging assessment standards was 100%, whereas compliance with the anatomically precise terminology standard was 0%. Table 2 summarises compliance with the five audit criteria.

**Table 2:** Compliance With Audit Criteria

Audit Criterion	Assessable Cases	Compliance	Target
Transvaginal ultrasonography used as the primary investigation	5	5/5 (100%)	100%
Compatible anatomical features documented	5	5/5 (100%)	100%
Uncertain findings appropriately escalated	2	2/2 (100%)	100%
Anatomically precise terminology used in the final ultrasound conclusion	5	0/5 (0%)	100%
Individualised management documented	5	5/5 (100%)	100%

Management and outcomes

Following confirmation of the diagnosis, management decisions considered clinical stability, serum β‑hCG level and trend, ultrasound anatomy, fertility-preserving options, anticipated haemorrhage risk, anaesthetic requirements, and available surgical expertise.

Management comprised successful single-dose methotrexate in Patient A, 1 (20%); hysteroscopic MyoSure resection in Patient D, 1 (20%); and surgery initiated laparoscopically in Patients B, C, and E, 3 (60%). Patient B, 1 (20%), had previously received methotrexate, but serial β‑hCG monitoring did not show the expected decline. Laparoscopic excision and suturing were completed in Patients C and E, 2 (40%). In Patient B, 1 (20%), laparoscopic excision was followed by minilaparotomy to suture the uterine cornu and secure haemostasis because laparoscopic suturing expertise was unavailable. Management was individualised according to clinical findings, serum β‑hCG trends, pregnancy location, anticipated haemorrhage risk, fertility-preserving options, and available expertise. Surgical complications were recorded in 0 (0%) patients, and 5 (100%) patients had a hospital stay of two days or fewer. Patient-level management and outcomes are summarised in Table 3.

**Table 3:** Management and Outcomes

Patient	Management	Surgical/Clinical Findings	Complications	Length of Stay
A	Single-dose methotrexate	Serial β-hCG levels showed an appropriate decline; no surgery required	None recorded	1 day
B	Methotrexate followed by laparoscopy and minilaparotomy	β-hCG levels did not show the expected decline after methotrexate. Laparoscopic excision was undertaken, but minilaparotomy was required to suture the uterine cornu and secure haemostasis because laparoscopic suturing expertise was unavailable.	None recorded	2 days
C	Laparoscopic excision and suturing	Procedure completed laparoscopically; the uterine cornu was sutured to achieve haemostasis	None recorded	1 day
D	Hysteroscopic MyoSure resection	Successful hysteroscopic resection of the interstitial pregnancy	None recorded	1 day
E	Laparoscopic excision and suturing	Procedure completed laparoscopically; the uterine cornu was sutured to achieve haemostasis	None recorded	1 day

## Discussion

The principal finding of this audit was a clear terminology gap: anatomically precise terminology was used in none of the five final ultrasound conclusions, despite documented imaging findings compatible with interstitial ectopic pregnancy. Transvaginal ultrasonography was performed in all cases, and uncertain findings were appropriately escalated to the lead sonographer. Diagnosis was established on the first scan in three cases (60%), while two cases (40%) required repeat specialist assessment before confirmation. All five pregnancies were diagnosed before rupture; this favourable observation is descriptive and cannot be attributed to the terminology used, the diagnostic pathway, or any management approach. The documented anatomical features supporting localisation included an empty uterine cavity, a laterally located gestational sac, a thin surrounding myometrial mantle, and the interstitial line sign [3,5,7].

Transvaginal ultrasonography remains the primary imaging modality for suspected interstitial ectopic pregnancy [3,8]. When conventional findings are equivocal, three-dimensional ultrasound or magnetic resonance imaging may assist in defining the relationship between the gestational sac, uterine cavity, and surrounding myometrium and in distinguishing interstitial pregnancy from angular pregnancy or other pregnancies implanted near the uterotubal junction [1,5,8]. In this audit, neither modality was required because the two uncertain cases were resolved through repeat transvaginal ultrasonography and lead-sonographer review, resulting in full compliance with the escalation standard.

This terminology discrepancy is important because ESHRE recommends that tubal ectopic pregnancies be described according to their anatomical location, including interstitial, isthmic, or ampullary implantation [1]. Interstitial, cornual, and angular pregnancies differ in anatomical location, prognosis, and management considerations [1,4,5]. Standardised terminology may therefore improve clinical communication, documentation consistency, coding accuracy, audit reliability, and future research reporting [1,4].

Importantly, this audit did not demonstrate that the use of the term “cornual ectopic pregnancy” resulted in delayed diagnosis, inappropriate management, or adverse clinical outcomes. All pregnancies were recognised before rupture and were managed appropriately according to their clinical circumstances. The observed terminology discrepancy should therefore be interpreted as a documentation and nomenclature gap rather than evidence of patient harm. Nevertheless, terminology standardisation remains an important quality-improvement objective because inaccurate nomenclature may affect communication between clinicians, coding practices, and the quality of clinical datasets used for audit and research [1,4,5].

Management was individualised in all cases according to clinical presentation, serum β-hCG concentration, ultrasound findings, fertility-preserving considerations, anticipated haemorrhage risk, and available expertise. Treatment approaches included medical management with methotrexate, hysteroscopic management, and surgical intervention. Hysteroscopic treatment has been reported as a fertility-preserving option for carefully selected, clinically stable interstitial ectopic pregnancies when the gestation is accessible from the uterine cavity and appropriate expertise is available [9,10]. In this audit, a successful hysteroscopic MyoSure resection without a recorded complication was performed in one patient. However, the small number of patients included prevents comparison of treatment modalities or assessment of the effectiveness of any individual management strategy.

Limitations

This audit has several important limitations. First, only five cases were identified over a five-year period. Although this reflects the rarity of interstitial ectopic pregnancy, the small sample size substantially limits generalisability and prevents meaningful assessment of associations between diagnostic terminology and clinical outcomes. Second, the audit was retrospective and depended on routinely recorded clinical and ultrasound documentation. Consequently, undocumented clinical reasoning cannot reliably be distinguished from considerations that were not documented at the time of patient care. Third, the study was conducted within a single centre with established ultrasound expertise and access to specialist management options, which may limit applicability to other settings.

Furthermore, the absence of a comparator group prevents attribution of timely diagnosis or favourable short-term outcomes to the EPAU pathway itself. Finally, although the use of “cornual ectopic pregnancy” in the final ultrasound conclusion was identified in all cases, the audit was not designed to determine whether this terminology discrepancy directly affected management decisions or patient outcomes. The findings should therefore be interpreted as identifying a specific and measurable terminology gap rather than establishing a causal relationship between diagnostic terminology and clinical outcomes.

Strengths

Despite these limitations, the audit has several strengths. It employed predefined audit criteria derived from ESHRE, RCOG, and NICE guidance, assessed a clinically relevant and measurable quality standard, and provided a detailed review of patient-level management and outcomes. The consistent identification of a terminology discrepancy across all cases highlights a clear opportunity for local quality improvement and future re-audit following implementation of educational and reporting interventions.

## Conclusions

The principal finding of this five-case retrospective audit was 0% compliance with anatomically precise terminology: all final ultrasound conclusions used the term "cornual ectopic pregnancy" despite imaging findings compatible with "interstitial ectopic pregnancy". This represents a terminology and documentation gap rather than evidence of deficient diagnostic recognition or clinical management. By contrast, the audit demonstrated 100% compliance with transvaginal ultrasound assessment, documentation of compatible anatomical features, escalation of diagnostically uncertain findings, and documented individualisation of management. All five pregnancies were diagnosed before rupture, no major complications were recorded, and hospital stays were two days or fewer. These favourable short-term outcomes are descriptive observations from five retrospective cases and do not demonstrate the effectiveness or superiority of the diagnostic pathway or any management strategy.

Although this audit did not demonstrate that the use of the term "cornual ectopic pregnancy" resulted in delayed diagnosis, inappropriate treatment, or adverse clinical outcomes, consistent anatomical nomenclature remains important for clear clinical communication, accurate documentation, coding, audit activity, and future research reporting. Educational initiatives and standardised ultrasound reporting templates should reinforce the distinction between interstitial, cornual, and angular pregnancies. A re-audit following the implementation of these measures is recommended to assess improvement in terminology compliance and reporting consistency.
